# Expansion and Functional Divergence of Shaker K^+^ Channels in Bermudagrass Highlight CdKAT1.1 in Salt Tolerance

**DOI:** 10.3390/ijms27073020

**Published:** 2026-03-26

**Authors:** Dong-Li Hao, Jia Qu, Jun-Yi Zhai, Rui-Qi Zhang, Shu-Yan Xi, Xi Xiang, Rong-Rong Chen, Hai-Lin Guo, Jun-Qin Zong, Jing-Bo Chen

**Affiliations:** 1 The National Forestry and Grassland Administration Engineering Research Center for Germplasm Innovation and Utilization of Warm-Season Turfgrasses, Jiangsu Key Laboratory for Conservation and Utilization of Plant Resources, Institute of Botany, Jiangsu Province and Chinese Academy of Sciences (Nanjing Botanical Garden Mem. Sun Yat-Sen), Nanjing 210014, China; haodongli@jib.ac.cn (D.-L.H.); qujia2026a@163.com (J.Q.); gggg6079@163.com (J.-Y.Z.); zhangruiqi2026@163.com (R.-Q.Z.); xishuyan2026@163.com (S.-Y.X.); xiangxi202601@163.com (X.X.); chenrongrong@jib.ac.cn (R.-R.C.); guohailin@jib.ac.cn (H.-L.G.); zongjunqin@jib.ac.cn (J.-Q.Z.); 2 Sanya Nanfan Research Institute of Hainan University, Sanya 572025, China

**Keywords:** shaker K^+^ channels, genome-wide identification, qPCR, potassium uptake, salt tolerance, bermudagrass

## Abstract

Salt stress inhibits plant growth, requiring salt-tolerant genes for the development of resilient plants. A key tolerance mechanism is potassium/sodium homeostasis, governed by Shaker K^+^ channels. Given that Shaker K^+^ channels from salt-sensitive species have been extensively studied while their counterparts in salt-tolerant plants remain largely unexplored, this study investigates the evolution and function of these channels in salt-tolerant bermudagrass to address this knowledge gap. Genomic analysis identified 25 Shaker K^+^ channel genes, an expanded family relative to other species. Phylogenetics placed them into five groups (I–V), with groups I, II, III, and V expanded via segmental duplication. Salt stress response screening revealed that only CdKAT1.1 was rapidly upregulated. Functional assays in yeast demonstrated that both CdKAT1.1 and its closest homolog CdKAT1.2 improve potassium uptake and salt tolerance, but the enhancement from CdKAT1.1 was significantly greater. This work elucidates the expansion and functional divergence of Shaker K^+^ channels in bermudagrass. CdKAT1.1 emerges as a superior regulator of potassium efficiency and salt tolerance, making it a prime candidate for molecular breeding to improve plant resilience in saline-alkaline soils.

## 1. Introduction

Soil salinization poses a critical global challenge to food security and land sustainability, with far-reaching environmental and socio-economic repercussions [1]. Globally, salinity affects approximately 424 million hectares of topsoil (0–30 cm depth), with salt-affected areas expanding annually by 2 million hectares, intensifying soil degradation and agricultural risks [2]. The exploration of salt-tolerant genes holds significant importance for cultivating new salt-tolerant germplasm, thereby enhancing the utilization efficiency of saline-alkali land. The K^+^/Na^+^ ratio serves as a critical determinant of plant salt tolerance, a physiological process closely associated with Shaker K^+^ channel gene family members that mediate potassium flux. The Shaker K^+^ channels are phylogenetically classified into five distinct groups (I–V) [3,4]. AKT1 is a member of Group I and plays a role in mediating root potassium ion absorption. Knocking it out reduced plant salt tolerance by lowering potassium content and K^+^/Na^+^ ratio, while overexpression improved plant salt tolerance by increasing potassium content and K^+^/Na^+^ ratio [5,6,7]. KAT1 is a member of Group II, mainly expressed in leaves, mediating the influx of potassium ions into leaf guard cells. Its expression in yeast can significantly enhance cell salt tolerance [4]. AKT2/3 is a member of Group III and is expressed in both roots and leaves. It plays a role in absorbing or excreting potassium according to the level of cell membrane potential. Knocking out this gene can significantly reduce plant salt tolerance [8]. KAT3 is a member of Group IV, and although it does not have a direct potassium absorption/efflux function, it can form heterodimers with other potassium ion channels to regulate its ability to absorb potassium [9]. It is speculated that it can indirectly regulate plant salt tolerance by regulating potassium absorption. SKOR is a member of Group V, mediating the transport of potassium ions through root-shoot translocation. Overexpression/knockout of this gene regulated plant salt tolerance [10,11]. These collective findings underscore the pivotal role of Shaker K^+^ channels in regulating plant salt tolerance through potassium homeostasis.

With the rapid development of genome sequencing technology, more and more species’ genomes are being published. Furthermore, with the help of gene family analysis and functional identification techniques, Shaker K^+^ channels of more and more species have been studied. It is worth noting that these studies mainly focus on shaker K^+^ channels derived from salt-sensitive plants, such as Arabidopsis, rice, foxtail millet [12], peach [13], sweet potato [14], mung bean [15], pear [16], apple [16], strawberry [16], and Chinese cabbage [4]. In contrast, there have been few reports on Shaker K^+^ channels derived from salt-tolerant plants. The Shaker K^+^ channels in salt-tolerant plants may possess unique functional regulatory mechanisms adapted to their specific habitats. There are significant differences in the number of Shaker K^+^ channel members among different species: for example, Arabidopsis has 9 Shaker K^+^ channel members, while rice has 10 [4], foxtail millet has 10 [12], peach has 7 [13], sweet potato has 11 [14], mung bean has 8 [15], pear has 8 [16], apple has 10 [16], strawberry has 6 [16], and Chinese cabbage has 13 members [4]. There are substantial differences in the function towards K^+^ transport capacity and salt tolerance of homologous proteins. For example, after expression in potassium-deficient yeast, BrKAT1.1 exhibits stronger potassium absorption activity and salt tolerance than SsKAT1.1 [4]. EoKAT1 has stronger potassium absorption activity and salt tolerance than EoKAT2 [17]. The above results regarding differences in the number of Shaker K^+^ channel family members and the functional and regulatory patterns of homologous genes further confirm the necessity of conducting research on the Shaker K^+^ channel gene family in salt-tolerant plants, rather than primarily focusing on salt-sensitive plant Shaker K^+^ channel gene families, to broaden the understanding of plant Shaker K^+^ channel gene families. Furthermore, research on the Shaker K^+^ channel gene family in salt-tolerant plants holds significant importance for obtaining superior salt-tolerant genes and serving the creation of new salt-tolerant germplasm resources.

In this study, we focused on bermudagrass (*Cynodon dactylon*), a salt-tolerant grass widely used in the ecological restoration of saline-alkali soils [18,19]. Using its completed genome sequence [20], we conducted a comprehensive bioinformatics analysis to identify Shaker K^+^ channel family members, determine their phylogenetic relationships, map their chromosomal locations, and analyze intra- and interspecific collinearity. The expression patterns of these genes under salt stress were examined by quantitative real-time PCR (qPCR). Salt-induced candidates were then heterologously expressed in a potassium-uptake-deficient yeast strain to assess their potassium transport activity, monitored by growth complementation under potassium-limited conditions. The same genes were subsequently introduced into a salt-sensitive yeast strain to evaluate their role in salt tolerance through comparative growth assays under NaCl stress. Together, these analyses expand our understanding of the evolutionary and functional diversity of Shaker K^+^ channels in a salt-tolerant species and identify CdKAT1.1 as a promising genetic resource for improving salt tolerance in bermudagrass breeding programs.

## 2. Results

### 2.1. Identification and Phylogenetic Analysis of Shaker K^+^ Channels in Bermudagrass

**Figure 1:**
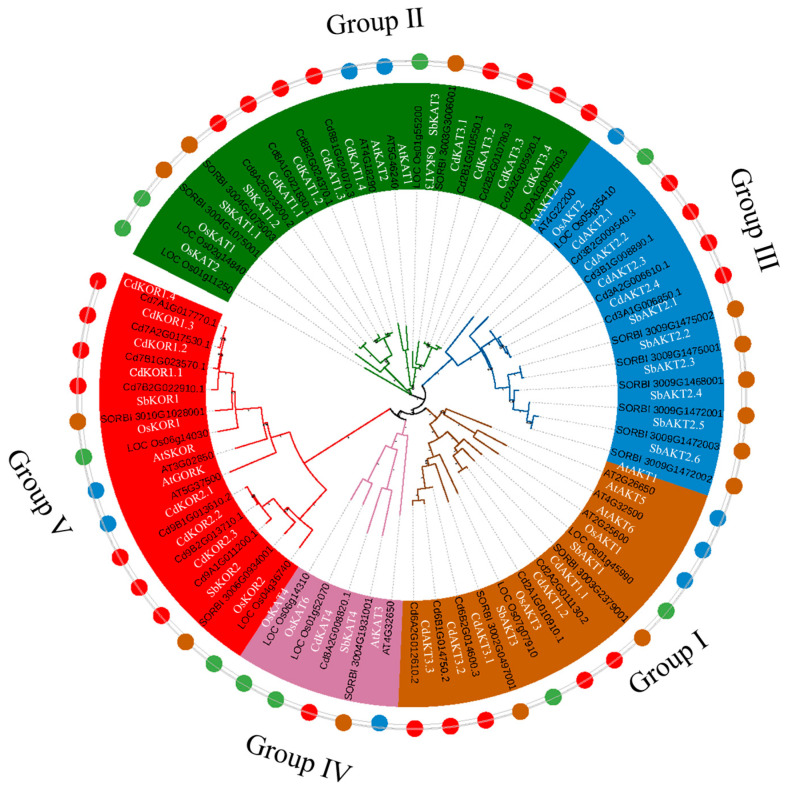
Phylogenetic tree of the relationships among the Shaker K^+^ channel proteins of bermudagrass, Arabidopsis, rice, and sorghum. Shaker K^+^ channel members from bermudagrass, Arabidopsis, rice, and sorghum are indicated by red dots, blue dots, green dots, and orange dots.

A total of 25 Shaker K^+^ channel members were identified in the bermudagrass genome. In comparison, 9 Shaker K^+^ channel members were identified in the Arabidopsis genome, 10 in the rice genome, and 14 in the sorghum genome (Figure 1). These results indicated that the bermudagrass genome harbors the highest number of Shaker K^+^ channel members. Phylogenetic analysis revealed that Shaker K^+^ channels from bermudagrass, Arabidopsis, rice, and sorghum could be classified into five groups (I–V). Members of Shaker K^+^ channels from all four species were distributed across all five groups. Regarding Arabidopsis, its group I contained 3 members (AtAKT1, AtAKT5, AtAKT6); group II included 2 members (AtKAT1, AtKAT2); group III had 1 member (AtAKT2/3); group IV comprised 1 member (AtKAT3); and group V contained 2 members (AtSKOR, AtGORK). Regarding rice, group I included 2 members (OsAKT1, OsAKT3); group II had 3 members (OsKAT1, OsKAT2, OsKAT3); group III contained 1 member (OsAKT2); group IV comprised 2 members (OsKAT4, OsKAT6); and group V included 2 members (OsKOR1, OsKOR2). Regarding to sorghum, its group I consisted of 2 members (SbAKT1, SbAKT3); group II contained 3 members (SbKAT1.1, SbKAT1.2, SbKAT3); group III had 6 members (SbAKT2.1, SbAKT2.2, SbAKT2.3, SbAKT2.4, SbAKT2.5, SbAKT2.6); group IV included 1 member (SbKAT4); and group V comprised 2 members (SbKOR1, SbKOR2). Regarding to bermudagrass, its group I contained 5 members (CdAKT1.1, CdAKT1.2, CdAKT3.1, CdAKT3.2, CdAKT3.3); group II included 8 members (CdKAT1.1, CdKAT1.2, CdKAT1.3, CdKAT1.4, CdKAT3.1, CdKAT3.2, CdKAT3.3, CdKAT3.4); group III comprised 4 members (CdAKT2.1, CdAKT2.2, CdAKT2.3, CdAKT2.4); group IV had 1 member (CdKAT4); and group V contained 7 members (CdKOR1.1, CdKOR1.2, CdKOR1.3, CdKOR1.4, CdKOR2.1, CdKOR2.2, CdKOR2.3). These findings demonstrated that, except for groups III and IV, which did not undergo gene expansion, significant gene expansion was observed in bermudagrass across the remaining three groups (I, II, and V).

The amino acid lengths encoded by bermudagrass Shaker K^+^ channel members were found to vary from 529 to 934 residues, with molecular weights spanning 60.54–105.97 kDa and isoelectric points (pI) ranging between 5.67 and 8.86 (Table 1). The shortest sequences were those of CdKAT3.1, CdKAT3.2, CdKAT3.3, and CdKAT3.4, whereas the longest sequence was encoded by CdAKT1.1. The lowest molecular weight was displayed by CdKAT4, while the highest molecular weight was associated with CdAKT1.1. Correspondingly, the most acidic pI value was observed for CdKAT4, and the most basic pI was noted for CdAKT1.1. Differences in amino acid length, molecular weight, and pI were evident both across groups and within members of the same group, implying that distinct Shaker K^+^ channel members may fulfill diverse functional roles.

### 2.2. Structural Analysis of Shaker K^+^ Channel Genes in Bermudagrass

**Figure 2:**
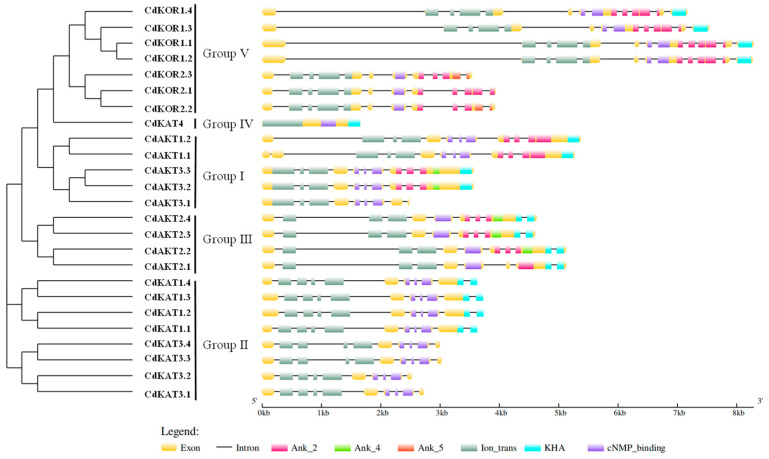
Phylogenetic tree and Protein domain among bermudagrass Shaker K^+^ channel family. The left panel shows a phylogenetic tree, and the right panel shows a protein domain result. Gray rectangles: Represent exons, the core coding regions that are translated into proteins. Thin black lines: Represent introns, the non-coding intervening sequences located between exons. Colored blocks: Represent different conserved functional domains. Pink: Ank_2 (Ankyrin repeat domain 2). Yellow-green: Ank_4 (Ankyrin repeat domain 4). Orange: Ank_5 (Ankyrin repeat domain 5). Purple: Ion_trans (Ion transport domain). Cyan: KHA (Potassium channel associated domain). Small purple blocks: cNMP_binding (Cyclic nucleotide monophosphate binding domain).

Gene structure analysis revealed that all groups except Group IV contained members with both exons and introns (Figure 2). In Group I, CdAKT1.1 comprised 12 exons and 11 introns, CdAKT1.2 had 11 exons and 10 introns, CdAKT3.1 contained 9 exons and 8 introns, and both CdAKT3.2 and CdAKT3.3 featured 10 exons and 9 introns. Group II members included four CdKAT1 genes (CdKAT1.1–CdKAT1.4), each with 11 exons and 10 introns, and four CdKAT3 genes (CdKAT3.1–CdKAT3.4), each with 10 exons and 9 introns. In Group III, CdAKT2.1 possessed 9 exons and 8 introns, while CdAKT2.2, CdAKT2.3, and CdAKT2.4 each contained 10 exons and 9 introns. The sole Group IV member, CdKAT4, lacked introns. Group V consisted of four CdKOR1 genes (CdKOR1.1–CdKOR1.4) with 12 exons and 11 introns and three CdKOR2 genes (CdKOR2.1–CdKOR2.3) with 11 exons and 10 introns. Overall, Shaker K^+^ channel members exhibited minimal variation in exon-intron numbers, with structural differences primarily arising from exon-intron spacing.

It was revealed through protein domain analysis that distinct domain compositions existed across Shaker K^+^ channel groups. For Group I members: CdAKT1.1 and CdAKT1.2 were characterized by the presence of Ion-trans, cNMP-binding, Ank2, and KHA domains; CdAKT3.1 was identified to contain Ion-trans and cNMP-binding domains, while CdAKT3.2 and CdAKT3.3 were found to harbor Ion-trans, cNMP-binding, Ank2, Ank4, and KHA domains. For Group II members: the four CdKAT1 members (CdKAT1.1–CdKAT1.4) were observed to contain Ion-trans, cNMP-binding, and KHA domains, whereas the four CdKAT3 members (CdKAT3.1–CdKAT3.4) were detected to carry only Ion-trans and cNMP-binding domains. In Group III: CdAKT2.1 was noted to include Ion-trans, cNMP-binding, Ank2, and KHA domains, while CdAKT2.2, CdAKT2.3, and CdAKT2.4 were documented to possess Ion-trans, cNMP-binding, Ank2, Ank4, and KHA domains. For Group IV: the sole member CdKAT4 was confirmed to exhibit Ion-trans, cNMP-binding, and KHA domains. In Group V: all four CdKOR1 members (CdKOR1.1–CdKOR1.4) were identified to contain Ion-trans, cNMP-binding, Ank2, and KHA domains. CdKOR2.1 was observed to feature Ion-trans, cNMP-binding, and Ank2 domains, while CdKOR2.2 and CdKOR2.3 were found to harbor Ion-trans, cNMP-binding, Ank2, and Ank5 domains. These results demonstrated that protein domain compositions varied not only between groups but also among members within the same group. This divergence in domain architecture was suggested to reflect potential functional or regulatory differences among Shaker K^+^ channel members.

### 2.3. Chromosomal Localization

**Figure 3:**
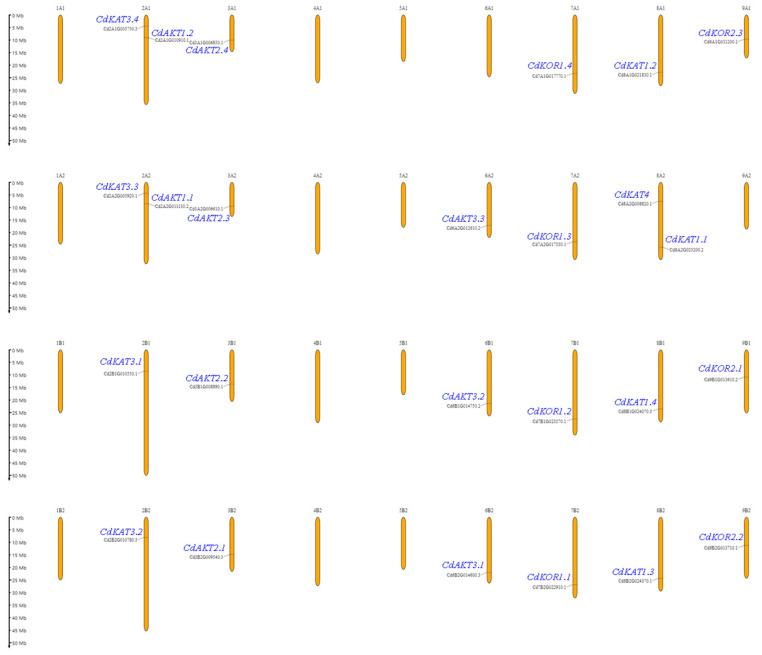
Position of Shaker K^+^ channel members on the chromosomes. The name and gene lD of the Shaker K^+^ channel are indicated.

The 25 Shaker K^+^ channel members were mapped to 21 out of the 36 pseudo chromosomes, while no members were identified on the remaining 15 pseudo chromosomes (Figure 3). Specifically, two Shaker K^+^ channels were localized to each of the following chromosomes: chr 2A1 (CdKAT3.4, CdAKT1.2), chr 2A2 (CdKAT3.3, CdAKT1.1), and chr 8A2 (CdKAT4, CdKAT1.1). One Shaker K^+^ channel was detected on each of the following chromosomes: chr 3A1 (CdAKT2.4), chr 7A1 (CdKOR1.4), chr 8A1 (CdKAT1.2), chr 9A1 (CdKOR2.3), chr 3A2 (CdAKT2.3), chr 6A2 (CdAKT3.3), chr 7A2 (CdKOR1.3), chr 2B1 (CdKAT1.3), chr 3B1 (CdAKT2.2), chr 6B1 (CdAKT3.2), chr 7B1 (CdKOR1.2), chr 8B1 (CdKAT1.4), chr 9B1 (CdKOR2.1), chr 2B2 (CdKAT3.2), chr 3B2 (CdAKT2.1), chr 6B2 (CdAKT3.1), chr 7B2 (CdKOR1.1), chr 8B2 (CdKAT1.3), and chr 9B2 (CdKOR2.2). These findings demonstrate an uneven chromosomal distribution of Shaker K^+^ channel members in bermudagrass.

### 2.4. Intraspecific Collinearity and Interspecific Collinearity

**Figure 4:**
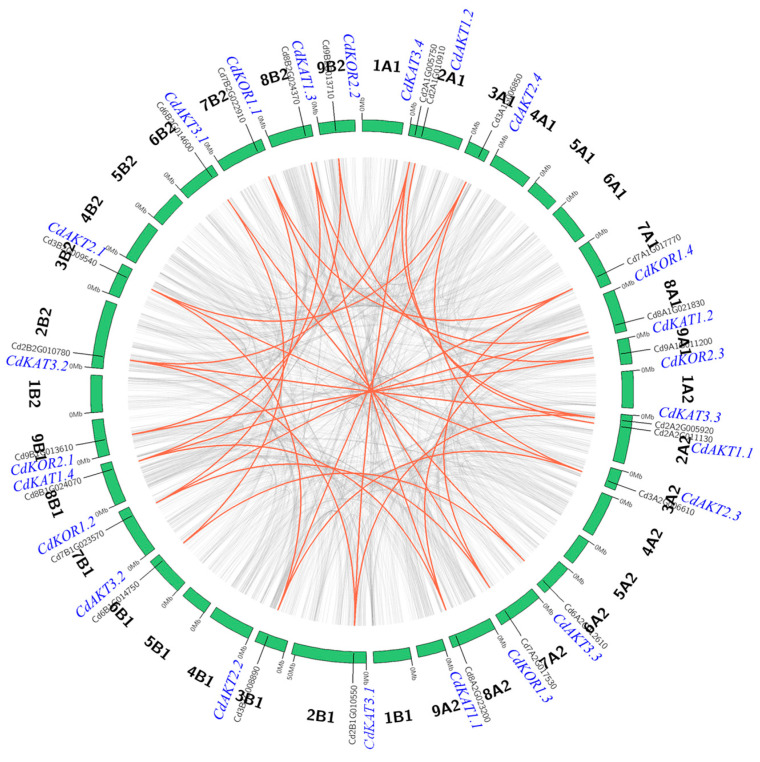
Intra-species collinearity analysis. The gray line represents the collinear gene pairs, whereas the red line represents the collinear Shaker K^+^ channel gene pairs in the genome of bermudagrass. The gene name (blue) and gene ID number are marked at the corresponding positions.

Intraspecific collinearity analysis revealed a substantial number of collinear gene pairs among bermudagrass genes (Figure 4). Within the Shaker K^+^ channel members, 31 collinear gene pairs were identified. Group I contained four pairs: CdAKT3.3-CdAKT3.2, CdAKT3.3-CdAKT3.1, CdAKT3.2-CdAKT3.1, and CdAKT1.2-CdAKT1.1. Group II exhibited twelve pairs, including CdKAT3.4-CdKAT3.3, CdKAT3.4-CdKAT3.1, CdKAT3.4-CdKAT3.2, CdKAT3.3-CdKAT3.1, CdKAT3.3-CdKAT3.2, CdKAT3.1-CdKAT3.2, CdKAT1.2-CdKAT1.1, CdKAT1.2-CdKAT1.4, CdKAT1.2-CdKAT1.3, CdKAT1.1-CdKAT1.4, CdKAT1.1-CdKAT1.3, and CdKAT1.4-CdKAT1.3. Group III comprised six pairs: CdAKT2.4-CdAKT2.3, CdAKT2.4-CdAKT2.2, CdAKT2.4-CdAKT2.1, CdAKT2.3-CdAKT2.2, CdAKT2.3-CdAKT2.1, and CdAKT2.2-CdAKT2.1. No collinear pairs were detected in Group IV. Group V featured nine pairs: CdKOR2.3-CdKOR2.1, CdKOR2.3-CdKOR2.2, CdKOR2.1-CdKOR2.2, CdKOR1.4-CdKOR1.3, CdKOR1.4-CdKOR1.2, CdKOR1.4-CdKOR1.1, CdKOR1.3-CdKOR1.2, CdKOR1.3-CdKOR1.1, and CdKOR1.2-CdKOR1.1.

**Figure 5:**
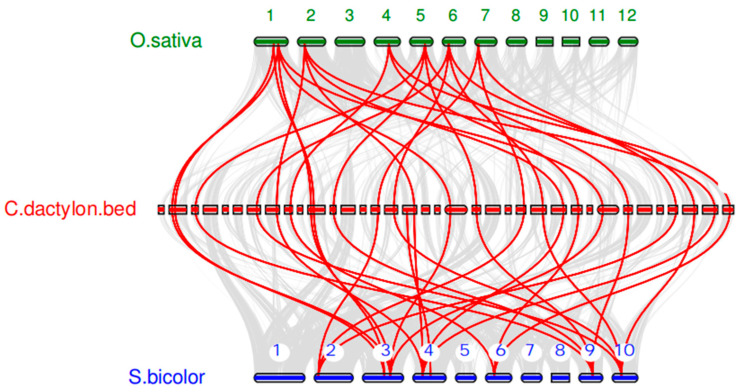
Collinearity analyses of Shaker K^+^ channel genes among bermudagrass, rice, and sorghum. The gray lines among the three plants represent collinear blocks in wide regions of the genome, whereas the red lines represent the orthologous relationships of the Shaker K^+^ channel genes.

The results of interspecific collinearity analysis (Figure 5) revealed that a large number of collinear gene pairs were identified among the genomes of bermudagrass, rice, and sorghum, with a greater number of collinear gene pairs being detected between bermudagrass and sorghum than between bermudagrass and rice. For the Shaker K^+^ channels, 25 collinear gene pairs were identified between bermudagrass and sorghum. In Group I, five collinear gene pairs were identified: SbAKT3-CdAKT3.3, SbAKT3-CdAKT3.2, SbAKT3-CdAKT3.1, SbAKT1-CdAKT1.2, and SbAKT1-CdAKT1.1. In Group II, eight collinear gene pairs were detected: SbKAT3-CdKAT3.4, SbKAT3-CdKAT3.3, SbKAT3-CdKAT3.1, SbKAT3-CdKAT3.2, SbKAT1.1-CdKAT1.2, SbKAT1.1-CdKAT1.1, SbKAT1.1-CdKAT1.4, and SbKAT1.1-CdKAT1.3. In Group III, four collinear gene pairs were observed: SbAKT2.2-CdAKT2.4, SbAKT2.2-CdAKT2.3, SbAKT2.2-CdAKT2.2, and SbAKT2.2-CdAKT2.1. In Group IV, one collinear gene pair was identified: SbKAT4-CdKAT4. In Group V, seven collinear gene pairs were revealed: SbKOR2-CdKOR2.3, SbKOR2-CdKOR2.1, SbKOR2-CdKOR2.2, SbKOR1-CdKOR1.4, SbKOR1-CdKOR1.3, SbKOR1-CdKOR1.2, and SbKOR1-CdKOR1.1. Between bermudagrass and rice, 24 collinear gene pairs were identified. In Group I, five collinear gene pairs were detected: OsAKT1-CdAKT1.2, OsAKT1-CdAKT1.1, OsAKT3-CdAKT3.3, OsAKT3-CdAKT3.2, and OsAKT3-CdAKT3.1. In Group II, eight collinear gene pairs were observed: OsKAT3-CdKAT3.4, OsKAT3-CdKAT3.3, OsKAT3-CdKAT3.1, OsKAT3-CdKAT3.2, OsKAT1-CdKAT1.2, OsKAT1-CdKAT1.1, OsKAT1-CdKAT1.4, and OsKAT1-CdKAT1.3. In Group III, four collinear gene pairs were identified: OsAKT2-CdAKT2.4, OsAKT2-CdAKT2.3, OsAKT2-CdAKT2.2, and OsAKT2-CdAKT2.1. No collinear gene pairs were detected in Group IV. In Group V, seven collinear gene pairs were found: OsKOR2-CdKOR2.3, OsKOR2-CdKOR2.1, OsKOR2-CdKOR2.2, OsKOR1-CdKOR1.4, OsKOR1-CdKOR1.3, OsKOR1-CdKOR1.2, and OsKOR1-CdKOR1.1. With the exception of KAT4, the remaining six Shaker K^+^ channels in bermudagrass were found to possess collinear gene pairs with both rice and sorghum, suggesting that these gene members may represent conserved genes during the evolutionary process of Poaceae. The collinear gene pair involving KAT4 was exclusively identified between bermudagrass and sorghum but not between bermudagrass and rice, indicating that this gene underwent an evolutionary process shared with sorghum but distinct from that of rice.

### 2.5. Promoter Analysis

**Figure 6:**
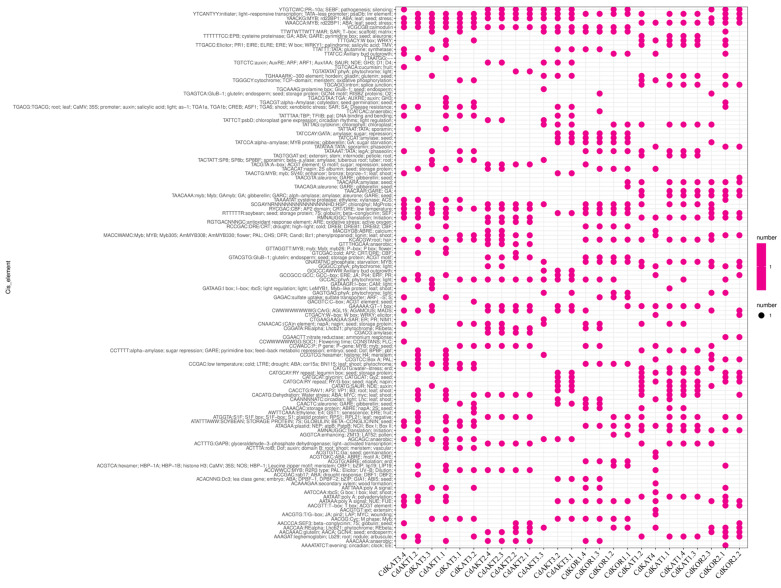
Promoter analysis of Shaker K^+^ channels in bermudagrass. The various regulatory elements upstream 2000 bp of the initiator codon of these genes were represented.

Promoter analysis revealed that multiple regulatory elements were identified on the promoters of bermudagrass Shaker K^+^ channels, covering processes such as light response, hormone regulation, pathogen infection, flowering, and other growth-related or stress-responsive pathways (Figure 6). In Group I, the promoter regulatory elements were quantified as follows: 44 for CdAKT1.1, 38 for CdAKT1.2, 32 for CdAKT3.1, 32 for CdAKT3.2, and 21 for CdAKT3.3. In Group II, the promoter regulatory elements were identified as follows: 35 for CdKAT1.1, 36 for CdKAT1.2, 31 for CdKAT1.3, 34 for CdKAT1.4, 26 for CdKAT3.1, 32 for CdKAT3.2, 24 for CdKAT3.3, and 31 for CdKAT3.4. In Group III, the promoter regulatory elements were observed as follows: 27 for CdAKT2.1, 28 for CdAKT2.2, 31 for CdAKT2.3, and 31 for CdAKT2.4. In Group IV, 28 promoter regulatory elements were detected for CdKAT4. In Group V, the promoter regulatory elements were recorded as follows: 32 for CdKOR1.1, 30 for CdKOR1.2, 34 for CdKOR1.3, 32 for CdKOR1.4, 39 for CdKOR2.1, 32 for CdKOR2.2, and 29 for CdKOR2.3. These results indicated that distinct promoter response elements were observed among different groups, and substantial differences in promoter response elements were also identified among members within the same group.

### 2.6. Expression Patterns of Shaker K^+^ Channel Genes in Response to Salt Stress

**Figure 7:**
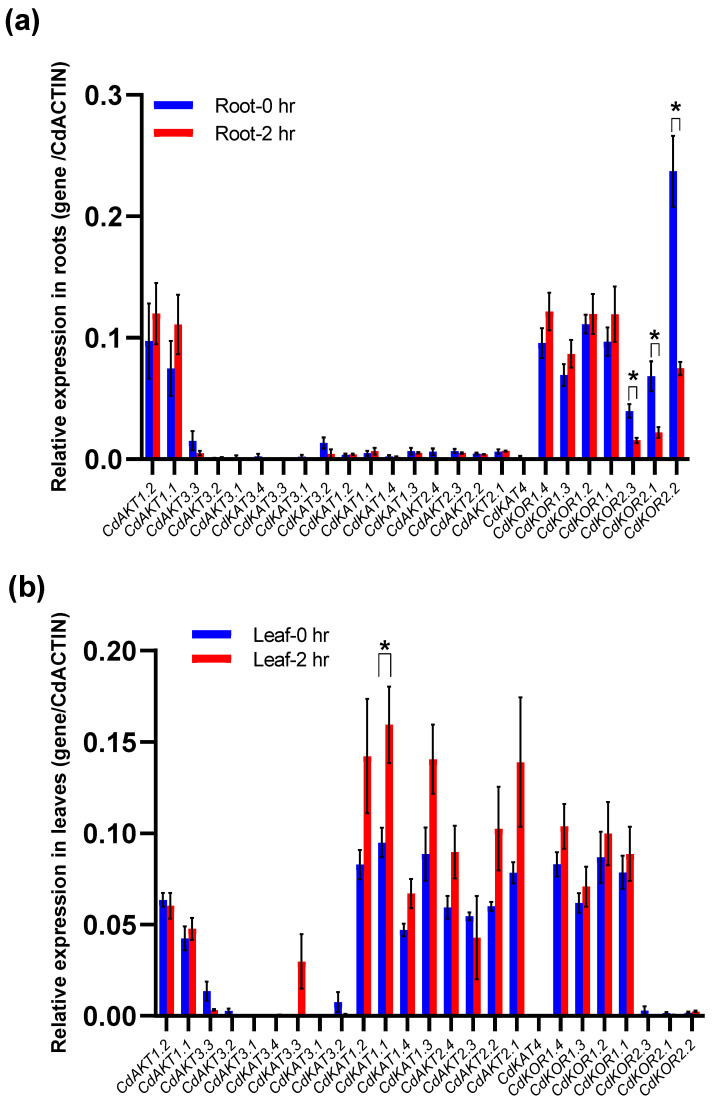
Expression profiles of Shaker K^+^ channel genes in bermudagrass under salt stress. (**a**,**b**) Relative expression levels of the genes in roots (**a**) and leaves (**b**) at 0 and 2 h after salt treatment, normalized to the reference gene CdACTIN. Genes showing statistically significant differences before and after treatment are marked with asterisks (*p* < 0.05, Student’s *t*-test); those without significant differences are unmarked.

Analysis of the expression patterns of 25 Shaker K^+^ channel genes revealed distinct profiles among them, mainly differing in relative expression levels and tissue-specific preferences (Figure 7). Under salt treatment, root gene responses followed two patterns: no significant change in expression (22 genes) or downregulation (3 genes: CdKOR2.1–CdKOR2.3). Notably, no Shaker K^+^ channel gene showed upregulated expression in roots under salt stress (Figure 7a). In leaves, salt treatment also induced two response patterns: most genes exhibited no significant expression change (24 genes), while one gene (CdKAT1.1) was upregulated (Figure 7b). These results indicate that CdKAT1.1 is the only gene among the 25 Shaker K^+^ channel genes that is rapidly upregulated under salt stress.

### 2.7. Functional Validation in Potassium Uptake-Deficient Yeast

**Figure 8:**
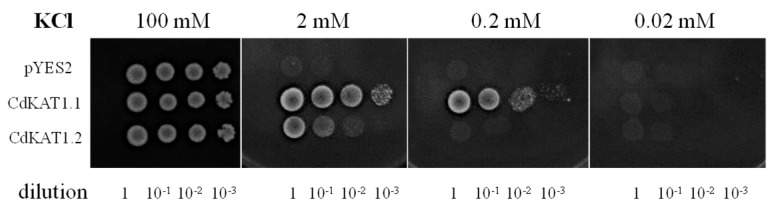
Yeast functional complementation. The growth of yeast mutant (deficient in K^+^ uptake) harbouring bermudagrass Shaker K^+^ channel under different concentrations of potassium is presented. The K^+^ concentration used is 0.02, 0.2, 2, and 100 mM. The dilution factor of the cell suspension is indicated.

Given that CdKAT1.1 is the only Shaker K^+^ channel gene among the 25 that is rapidly upregulated under salt stress (Figure 7), we assessed its potassium uptake activity using a potassium uptake-deficient yeast strain. Its highly homologous gene, CdKAT1.2, was included as a parallel control (Figure 8). Under sufficient potassium supply (100 mM), it was observed that both yeast expressing Shaker K^+^ channel genes and yeast transformed with empty vectors grew normally, with no significant differences in growth performance between them, indicating that the introduction of different Shaker K^+^ channel genes had no impact on yeast growth. Under restricted potassium supply (≤2 mM), growth failure was exhibited by yeast transformed with empty vectors, consistent with the characteristic of this potassium uptake-deficient yeast strain [4,12,21]. Distinct growth response patterns were displayed by yeast transformed with CdKAT1.1 and CdKAT1.2. Complete restoration of growth in potassium uptake-deficient yeast under 2 mM K^+^ supply was achieved by CdKAT1.1, whereas only partial restoration was observed for CdKAT1.2. This phenomenon was maintained when external potassium concentration was further reduced to 0.2 mM, demonstrating that stronger potassium uptake activity was possessed by CdKAT1.1 compared to CdKAT1.2. Gradual enhancement of complementation effects was shown by yeast transformed with CdKAT1.1 and CdKAT1.2 as external potassium concentrations increased, indicating that potassium absorption by both channels was concentration-dependent.

### 2.8. Functional Validation in Salt-Sensitive Yeast

**Figure 9:**
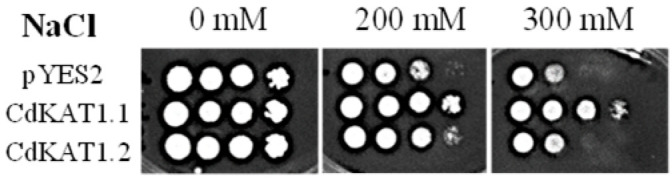
Expression of CdKAT1.1 and CdKAT1.2 in a salt-sensitive yeast mutant. The growth of CdKAT1-expressing yeast mutant (G19, a salt-sensitive yeast strain) on the AP medium supplemented with different concentrations of NaCl and a fixed concentration of KCl (4 mM) is presented. The dilution factor of the cell suspension is indicated.

To investigate their roles in cellular salt tolerance, CdKAT1.1 and CdKAT1.2—two potassium-absorbing genes—were introduced into the salt-sensitive yeast strain G19 (Figure 9). Yeast transformed with CdKAT1.1, CdKAT1.2, or empty vectors exhibited comparable growth on NaCl-free medium, confirming that neither gene disrupted normal yeast growth. Consistent with prior studies [22], yeast carrying empty vectors showed progressive growth inhibition as NaCl concentrations increased. Under 200 mM NaCl stress, both CdKAT1.1- and CdKAT1.2-transformed yeast outperformed empty vector controls in growth, though CdKAT1.1-transformed yeast displayed substantially stronger salt tolerance than CdKAT1.2-transformed yeast. This disparity became more evident at 300 mM NaCl. Together, these findings demonstrate that both genes enhance yeast salt tolerance, with CdKAT1.1 conferring markedly greater resilience than CdKAT1.2.

## 3. Discussion

### 3.1. Bermudagrass Exhibits an Obvious Gene Expansion in Its Shaker K^+^ Channels Compared to Other Species

Genome-wide identification revealed 25 Shaker K^+^ channel members in bermudagrass (Figure 1), all of which retain conserved Shaker K^+^ channel protein domains and exhibit an uneven chromosomal distribution (Figure 2 and Figure 3). Importantly, the total number of Shaker K^+^ channel genes in bermudagrass exceeds that reported in most other plant species (Table S1), with a particularly striking feature being the pronounced expansion of Groups II and V (8 and 7 members, respectively). This expansion is a lineage-specific trait, as no other species analyzed to date possesses more than 4 members in either of these two groups (Table S1). Bermudagrass maintains intermediate gene counts in Groups I (5) and III (4), while Group IV remains minimally represented (1), indicating that the overall expansion of the Shaker K^+^ channel family in this species is primarily attributed to the proliferation of Groups I, II, III, and V. Collinearity analysis within bermudagrass identified segmental duplication among these groups as the primary mechanism driving family expansion (Figure 4), and the uniform distribution of collinear gene pairs across four chromosome sets further implies evolutionary associations with genome duplication events [19,20,23]. Building on the well-established role of Shaker K^+^ channels in mediating plant salt tolerance [6,8,11], the expansion of this gene family in bermudagrass is likely an adaptive evolutionary strategy that enhances its resilience and adaptation to saline-alkali environments, providing novel insights into the functional diversification of Shaker K^+^ channels in grasses.

### 3.2. CdKAT1.1 Plays a Critical Role in Salt Tolerance of Bermudagrass

Genome-wide identification revealed 25 Shaker K^+^ channel members in bermudagrass (Figure 1). CdKAT1.1 was the only Shaker K^+^ channel gene among the 25 examined that was rapidly upregulated under salt stress (Figure 7). Functional characterization further revealed that CdKAT1.1 confers remarkably strong potassium uptake activity and salt tolerance—significantly surpassing that of its highly homologous counterpart, CdKAT1.2 (Figure 8 and Figure 9). Together, these results demonstrate that CdKAT1.1 plays a key role in the salt tolerance of bermudagrass, making it a promising target gene for breeding highly salt-tolerant bermudagrass varieties.

Although numerous studies have reported on plant Shaker K^+^ channel genes in response to salt stress, most have focused only on isolated individual or a few members [8,24,25], with a lack of reports systematically examining the salt-responsive expression patterns across the entire Shaker K^+^ channel family. In this study, we comprehensively report the salt-stress expression patterns of all 25 members of the Shaker K^+^ channel family in different bermudagrass tissues, thereby enriching the understanding of this gene family’s transcriptional response to salt stress. While several Shaker K^+^ channel genes in rice are upregulated under salt stress [22], only one such gene is induced in bermudagrass, which may reflect species-specific differences in how Shaker K^+^ channels respond to salt stress. It is worth noting that this study was conducted using only a single bermudagrass accession. Future gene expression analyses under salt stress using multiple bermudagrass germplasm resources would be more effective in confirming the key role of CdKAT1.1 in specifically responding to salt stress.

### 3.3. Functional Divergence Among Shaker K^+^ Channels

Both CdKAT1.1 and CdKAT1.2 were confirmed to possess potassium absorption activity (Figure 8), consistent with the conserved functions reported for their homologs in other species [26,27,28]. However, a marked disparity in uptake capacity was observed, with CdKAT1.1 exhibiting significantly stronger absorption than CdKAT1.2 (Figure 8). Given that enhanced potassium uptake is known to improve cellular salt tolerance by optimizing the K^+^/Na^+^ ratio, the superior salt tolerance conferred by CdKAT1.1 over CdKAT1.2 (Figure 9) further supports this mechanism. Notably, this pronounced functional difference between CdKAT1.1 and CdKAT1.2 contrasts with observations in other species—such as AtKAT1/AtKAT2 in Arabidopsis, OsKAT1/OsKAT2 in rice, and BrKAT1.1/BrKAT1.2 in Chinese cabbage—where homologous pairs generally show comparable or only slightly divergent uptake activities [4,29,30,31]. This distinction may represent a species-specific adaptation in bermudagrass.

Despite their collinear evolutionary relationship (Figure 4), CdKAT1.1 and CdKAT1.2 exhibit clear functional divergence in promoter response elements, salt-stress expression patterns, potassium uptake activity, and salt tolerance (Figure 6, Figure 7, Figure 8 and Figure 9). This divergence is likely an adaptive trait that enables differentiated responses to environmental stress.

## 4. Materials and Methods

### 4.1. Genome-Wide Identification of Shaker K^+^ Channel Family Members

Following our previous methods [32], the Pfam_scan program (version 14.0, https://github.com/SMRUCC/GCModeller/tree/master/src/interops/scripts/PfamScan, accessed on 20 October 2023) was used to search against the Pfam 35.0 database at the whole-proteome level using the predicted protein sequences of bermudagrass (*Cynodon dactylon*), Arabidopsis (*Arabidopsis thaliana*), rice (*Oryza sativa*), and sorghum (*Sorghum bicolor*). The analysis was performed with a strict E-value threshold ≤ 1 × 10^−5^ and default bit-score cutoff as implemented in Pfam_scan. Sequences containing both of the conserved Shaker K^+^ channel domains (PF00027.28: cNMP_binding and PF00520.30: Ion_trans) were initially retrieved. Subsequent filtering steps were applied to ensure accuracy: (i) only sequences encoding both core domains were retained; (ii) partial sequences (length < 60% of typical full-length Shaker channels) were excluded; (iii) redundant isoforms with ≥95% sequence identity were removed, retaining only the longest representative for each locus. This analysis revealed 25 Shaker K^+^ channel family members in bermudagrass, 9 in Arabidopsis, 10 in rice, and 14 in sorghum. Genome/proteome data sources were as follows: Bermudagrass: https://figshare.com/s/4afb40bda6b43e6e37a4 (accessed on 25 June 2022). Arabidopsis: https://www.ncbi.nlm.nih.gov/datasets/genome/GCF_000001735.4/ (accessed on 23 August 2023). Rice: https://www.ncbi.nlm.nih.gov/datasets/genome/GCF_001433935.1/ (accessed on 23 August 2023). Sorghum: https://www.ncbi.nlm.nih.gov/datasets/genome/GCF_000003195.3/ (accessed on 23 August 2023).

### 4.2. Phylogenetic Tree Analysis, Gene Structure, and Chromosome Position

Phylogenetic tree analysis: Global alignment among the Shaker K^+^ channel members identified in all four species was conducted using the MUSCLE program (version 5.0, https://drive5.com/muscle/ (accessed on 23 August 2023)). Based on this alignment, the phylogenetic tree shown in Figure 1 was constructed using the maximum likelihood (ML) method with 1000 bootstrap replicates in the MEGA program (version 11, https://www.megasoftware.net/ (accessed on 23 August 2023)). The phylogenetic tree was visualized using the iTOL program (version 5.0, https://itol.embl.de/ (accessed on 23 August 2023)) [4].

Gene structure: Motif information of the Shaker K^+^ channels was extracted using the Pfam annotation results, and a motif distribution map was generated with the GSDS program (version 2.0, http://gsds.gao-lab.org/, accessed on 10 December 2023) [4].

Chromosome position: Information on Shaker K^+^ channels was extracted from the GFF file of the bermudagrass genomic database. The positions of these genes were visualized using the MG2C program (version 2.1, http://mg2c.iask.in/mg2c_v2.1/ (accessed on 23 August 2023)) [33].

### 4.3. Intraspecific Collinearity and Interspecific Collinearity

Intraspecific collinearity: First, collinear blocks in the bermudagrass genome were identified using the MCScanX program (version 2.0, https://github.com/wyp1125/MCScanx (accessed on 23 August 2023)). Subsequently, Shaker K^+^ channel members located within these collinear blocks were extracted. Finally, visualization of the intraspecific collinearity results was performed using the Circos program (version 0.69, http://circos.ca/ (accessed on 23 August 2023)) [34].

Interspecific Collinearity: Collinear blocks among the bermudagrass genome, sorghum genome, and rice genome were identified using the JCVI program (version 0.9.13, https://github.com/tanghaibao/jcvi (accessed on 23 August 2023)). Following this, Shaker K^+^ channel members located within these collinear blocks were extracted. Visualization of the interspecific collinearity results was carried out using the JCVI program (version 0.9.13, https://github.com/tanghaibao/jcvi (accessed on 23 August 2023)) [35].

### 4.4. Promoter Analysis

For each bermudagrass shaker K^+^ channel member, the promoter region was identified as a 2000 bp sequence upstream of the ATG translational initiation site. Subsequently, cis-acting regulatory elements are predicted using the PlantCARE tool (http://bioinformatics.psb.ugent.be/webtools/plantcare/html/ (accessed on 23 August 2023)). Finally, the visualization of the promoter analysis results is taken using the TBtools software, version 1.120 [4].

### 4.5. Plant Culture and Gene Expression Profile Analysis

The plant culture procedure was conducted following an established protocol [32]. Briefly, stolons with three nodes were initially cultured in water for seven days to promote root emergence. Uniform plants were then selected and cultivated hydroponically for fourteen days using a 1/2 Hoagland’s nutrient solution. Subsequently, the plants were subjected to salt treatments (0 and 200 mM NaCl). Two hours after treatment, roots and leaves were separately sampled. The collected samples were ground into a fine powder using liquid nitrogen. Total RNA was extracted with RNAiso (Takara, Kusatsu, Japan) and treated with RNase-free DNase I (Takara, Japan) for 15 min to remove potential contaminating DNA. RNA quality was assessed by agarose gel electrophoresis and by measuring the A260/A280 ratio. One microgram of total RNA was reverse-transcribed into cDNA using a PrimeScript RT kit (Takara, Japan). Gene expression analysis was performed via real-time quantitative PCR (qPCR) on a StepOne Real-Time PCR System (Applied Biosystems, Waltham, MA, USA). The qPCR protocol consisted of an initial step at 95 °C for 30 s, followed by 40 cycles of 95 °C for 5 s and 60 °C for 30 s. The raw qPCR data were collected using BioRad CFX Maestro software (version 2.3). Relative gene expression levels were calculated using the 2−ΔCt method [36], where the Ct values of target genes were first normalized to the internal reference gene (CdACTIN) to obtain ΔCt values for comparison. All treatments included three biological replicates. Primers were designed using Primer software (version 5.0), and their sequences are listed in Table 2. CdACTIN (gene ID: Cd4B2G007350.1) was used as the reference gene for normalization. Statistical significance was determined using a Student’s *t*-test in SPSS (version 26.0), with a significance level set at *p* < 0.05.

### 4.6. Gene Cloning and Vector Construction

After treatment, the whole plants were harvested. RNA was extracted using an RNA extraction kit (#RC201, Vazyme, Nanjing, China), and cDNA was synthesized using a reverse transcription kit (#R211, Vazyme, Nanjing, China). The PCR procedure for Shaker K^+^ channel cloning was carried out under the following conditions: initial denaturation at 95 °C for 5 min, followed by 30 cycles of denaturation (95 °C for 15 s), annealing (65 °C for 15 s), and extension (72 °C for 2 min), with a final extension at 72 °C for 5 min [4]. The verified CdKAT1.1 and CdKAT1.2 genes were inserted into the yeast expression vector pYES2. The primers used for CdKAT1.1 cloning are: F: actatagggaatattaagcttATGACTCGAGCTCATTCATACTCATGC and R: tgatggatatctgcagaattcCTACATCTGAAGAAGGAATAGGTGGTC. The primers used for CdKAT1.2 cloning are: F: actatagggaatattaagcttATGCATGTTCATTGCCTGAAGCATATAC and R: tgatggatatctgcagaattcCTACATCTGAAGAAGGAATAGGTGGTCA.

### 4.7. Yeast Assays

Yeast transformation was performed using the yeast transformation kit (#SK2400, Coolaber, Beijing, China). Yeast transformants harboring Shaker K^+^ channel genes, which were screened on SD-URA media, were verified through sequencing (General Biology, Chuzhou, China) [32].

The potassium ion absorption-deficient yeast strain R5421 (Coolaber, Beijing, China) was utilized for potassium uptake ability evaluation. Sequenced transformants were cultured in SD-URA liquid media supplemented with 50 mM KCl at 30 °C for 2 days. When the optical density at 600 nm (OD600) reached 0.6–0.8, cells were collected, and the OD600 was adjusted to 1.0 using sterile water. The suspension was subsequently diluted 10-fold, 100-fold, and 1000-fold. A volume of 5 microliters of the diluted suspension was spotted onto AP-URA solid medium supplemented with KCl at concentrations of 100, 2, 0.2, and 0.02 mM. Yeast cultures were then incubated at 30 °C, and photographs were taken two days post-inoculation [32].

The salt-sensitive yeast strain G19 (Baosai, Beijing, China) was employed for salt tolerance ability evaluation. Transformants expressing CdKAT1.1 and CdKAT1.2 were cultured in SG-URA liquid medium at 30 °C for 2 days. When the OD600 reached 0.6–0.8, cells were harvested and resuspended in sterile water to adjust the OD600 to 1.0. The suspension was subsequently diluted with sterile water to OD600 values of 0.1, 0.01, and 0.001. A 5 µL aliquot of the diluted suspension was spotted onto AP-URA solid medium supplemented with varying concentrations of NaCl (0, 200, and 300 mM) and a fixed concentration of KCl (4 mM). Yeast cultures were then incubated at 30 °C, and photographs were taken 2 days post-inoculation [4].

## 5. Conclusions

Bermudagrass shows a marked expansion in Shaker K^+^ channel genes compared with other species, driven mainly by segmental duplications across four groups. Since these channels are closely linked to salt tolerance, such expansion likely underlies bermudagrass adaptation to saline-alkali soils. Furthermore, the collinear homologs CdKAT1.1 and CdKAT1.2 exhibit functional divergence, potentially enabling distinct stress-response strategies. Between them, CdKAT1.1 represents a valuable target for genetically improving potassium use efficiency and salt tolerance in bermudagrass.

## Figures and Tables

**Table 1:** The basic information of bermudagrass Shaker K^+^ channels.

Gene Name	Length	MV (Da)	pI	Group
CdAKT1.2	885	100,447.2	7.73	I
CdAKT1.1	934	105,966.6	8.86	I
CdAKT3.3	896	99,743.4	7.69	I
CdAKT3.2	895	99,631.1	7.31	I
CdAKT3.1	568	63,999.5	8.33	I
CdKAT3.4	529	60,746.7	8.04	II
CdKAT3.3	529	60,762.8	8.04	II
CdKAT3.1	529	60,755.8	7.67	II
CdKAT3.2	529	60,681.7	7.69	II
CdKAT1.2	707	81,861.3	7.08	II
CdKAT1.1	671	77,577.3	6.71	II
CdKAT1.4	671	77,495.2	6.75	II
CdKAT1.3	705	81,473.8	6.96	II
CdAKT2.4	841	93,117.9	7.3	III
CdAKT2.3	841	93,084.9	7.42	III
CdAKT2.2	843	93,409.3	8.09	III
CdAKT2.1	789	87,722.1	8.71	III
CdKAT4	551	60,541.1	5.67	IV
CdKOR1.4	843	95,504.5	6.64	V
CdKOR1.3	846	96,072	6.39	V
CdKOR1.2	896	100,995.5	6.48	V
CdKOR1.1	914	102,902.9	6.66	V
CdKOR2.3	702	79,361.5	8.15	V
CdKOR2.1	694	78,627.8	8.26	V
CdKOR2.2	694	78,760.9	8.18	V

**Table 2:** Primers used for qPCR.

ID	Gene Name	Forward Primers (5′–3′)	Reverse Primers (5′–3′)
Cd2A1G010910.1	CdAKT1.2	CCGCTATGGGAGGCTATGTG	GCCGTGTCCCCAGATGATAG
Cd2A2G011130.2	CdAKT1.1	TGCAGAATGAAGCACCCACT	TGCTCCGCACCATTTTGTTG
Cd6A2G012610.2	CdAKT3.3	CTTCTACTACCTGCTCGCCG	TACATGGACGCGATGTACCG
Cd6B1G014750.2	CdAKT3.2	GGAGCCCTCTTTGCCAATCT	CGGCGAGCAGGTAGTAGAAG
Cd6B2G014600.3	CdAKT3.1	TCTTTGGCCTCGTCCAGAAC	CTCGGCGCAAAGTATTCAGC
Cd2A1G005750.3	CdKAT3.4	TGTACTCGGCGTGGATATGC	TGTCGACGTACGGGACAAAG
Cd2A2G005920.1	CdKAT3.3	CCTCTTCTTCCCGGTTGTCC	CCGCATCTCTGTCACCAGTT
Cd2B1G010550.1	CdKAT3.1	TCATGCCGGACTTCAAGGAC	TACCCCGTGGTAGTCATCGT
Cd2B2G010780.3	CdKAT3.2	ACTCTGTTCGCCATCCACTG	GGACGTCACGTACCGTATCC
Cd8A1G021830.1	CdKAT1.2	CAGAGCTGAGTGGCCAAGAA	CCAAGAGATGGCAGGAGCTC
Cd8A2G023200.2	CdKAT1.1	GCTGCAGAGGGAGACATGTT	CTTCCACGTGTTCACGCATC
Cd8B1G024070.3	CdKAT1.4	CCAACCAGCTCGAAGGTTCA	CAGGTATGGCTGTGGCAAGA
Cd8B2G024370.1	CdKAT1.3	CGAGCATTTCACCATCGAGC	AATGAGGAACAGCTCCCAGC
Cd3A1G006850.1	CdAKT2.4	CGCCACTCGACTCCAGATAC	TTCATGAACGCCACCTCGAA
Cd3A2G006610.1	CdAKT2.3	CTCCTTCAACCTCCGGAACC	GTATCTGGAGTCGAGTGGCG
Cd3B1G008890.1	CdAKT2.2	GGGGTGCCTCTACTACCTGA	TGGACCAGTAGACGGAGGAG
Cd3B2G009540.3	CdAKT2.1	AGCAGTTCTTCACCAGGCTC	TCAGGTAGTAGAGGCACCCC
Cd8A2G008820.1	CdKAT4	AGTTCGGGATTGCAGTGAGG	TGATCTCCATCACGTAGCGC
Cd7A1G017770.1	CdKOR1.4	GGTGAAGACGGCCAAGAAGA	GGTGAAGGACTGCTTGTCGA
Cd7A2G017530.1	CdKOR1.3	ACACATACAGCTGCCTGCAT	CCCCAACGTTAAACTCCCGA
Cd7B1G023570.1	CdKOR1.2	TGTGATCAGGCTGCAAGAGG	TCTTCTTGGCCGTCTTCACC
Cd7B2G022910.1	CdKOR1.1	TCGGGAGTTTAACGTTGGGG	TGACGGCATGAATGTCACCA
Cd9A1G011200.1	CdKOR2.3	ACCTTGCATGGATCTGTGGG	CATACCAATTCGCTGGCAGC
Cd9B1G013610.2	CdKOR2.1	GCGTCTTCTACTACCTCGCC	GCAAGTGAGCAGGTCCATCT
Cd9B2G013710.1	CdKOR2.2	TGCACTTTGTCGCCAACATG	GGGTGACCGCAACTGTTCTA
Cd4B2G007350.1	CdACTIN	AAGGAGATCACTGCCTTGGC	TCAGCCTTCGCAATCCACAT

## Data Availability

The original contributions presented in this study are included in the article/Supplementary Material. Further inquiries can be directed to the corresponding author.

## Supplementary Materials

The following supporting information can be downloaded at: https://www.mdpi.com/article/10.3390/ijms27073020/s1.
