# Supplementary material for: Expansion and Functional Divergence of Shaker K+ Channels in Bermudagrass Highlight CdKAT1.1 in Salt Tolerance
Source: Int J Mol Sci. 2026 Mar 26;27(7):3020. doi: 10.3390/ijms27073020 (PMC13073167; doi:10.3390/ijms27073020)
Supplement: Supplementary file 1 [file ijms-27-03020-s001.zip › ijms-4209127-supplementary.pdf]

Expansion and Functional Divergence of Shaker K^+ Channels in Bermudagrass Highlight CdKAT1.1 in Salt Tolerance

Dong-Li Hao[1], Jia Qu[1,3], Jun-Yi Zhai[1], Rui-Qi Zhang[1], Shu-Yan Xi[1], Xi Xiang[1], Rong-Rong Chen[1], Hai-Lin Guo[1], Jun-Qin Zong[1], Jing-Bo Chen[1,*]

Table S1. The numbers of Shaker K+ channels in different species.

**Species**	Group I	Group II	Group III	Group IV	Group V	Total number	References
**Arabidopsis**	3	2	1	1	2	9	Zhou et al., 2024
**rice**	2	3	1	2	2	10	Zhou et al., 2024
**sorghum**	2	3	6	1	2	14	Zhou et al., 2024
**cotton**	8	4	4	4	4	24	Wang et al., 2023
**Chinese cabbage**	3	4	1	2	3	13	Zhou et al., 2024
**sweet potato**	3	2	1	2	3	11	Jin et al., 2021
**Foxtail Millet**	2	3	1	2	2	10	Zhang et al., 2022b
**grapevine**	2	1	1	1	4	9	Cuellar et al., 2013
**pear**	2	1	1	1	2	7	Chen et al., 2019a
**peach**	2	1	1	1	2	7	Yang et al., 2022
**bermudagrass**	5	8	4	1	7	25	This study
